# Emerging Pathways of Action of Eicosapentaenoic Acid (EPA)

**DOI:** 10.1016/j.jacbts.2024.10.010

**Published:** 2025-03-24

**Authors:** Deepak L. Bhatt, Peter Libby, R. Preston Mason

**Affiliations:** a Mount Sinai Fuster Heart Hospital, Icahn School of Medicine at Mount Sinai, New York, NY, USA; b Department of Medicine, Cardiovascular Division, Brigham and Women’s Hospital, Harvard Medical School, Boston, Massachusetts, USA; c Elucida Research, Beverly, Massachusetts, USA

**Keywords:** eicosapentaenoic acid, inflammation, omega-3 fatty acids

**Figure:**
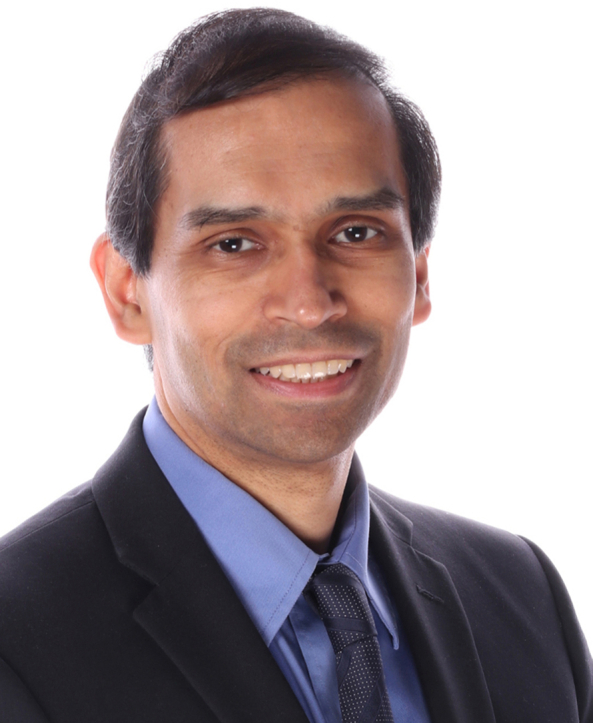

Substantial randomized data support the cardiovascular benefits of the omega-3 fatty acid eicosapentaenoic acid (EPA).^1^, ^2^, ^3^, ^4^ Yet, lack of a clear understanding of the likely multifactorial mechanism of benefit of EPA has generated skepticism among some physicians, potentially leading to underuse of this clinically beneficial, cost-effective, and safe therapy. The REDUCE-IT (Reduction of Cardiovascular Events with Icosapent Ethyl–Intervention Trial) showed large relative and absolute risk reductions in ischemic events with icosapent ethyl (a highly purified, pharmaceutical grade ethyl ester of EPA), including a significant reduction in cardiovascular death in secondary and high-risk primary prevention patients with mildly to moderately elevated triglycerides.^1^ Interestingly, the benefit extended across the full range of baseline triglyceride values in the trial, including in the subgroup of patients who entered the trial with normal triglyceride levels.^5^ Similarly, the benefit of icosapent ethyl extended to those patients who either did or did not achieve triglyceride levels within the normal range during the trial.^1^ Mediation analyses suggested that the triglyceride reduction accounted for a minority of the drug’s benefit, with approximately two-thirds of the benefit caused by the large increase in serum EPA that occurred on treatment. However, that observation does not actually explain how the EPA itself exerts its cardioprotective benefits.

In this issue of *JACC: Basic to Translational Science*, Reilly et al^6^ explore 1 possible mechanism of benefit of EPA—an anti-inflammatory effect. Studying nonactivated CD4+ T cells, the authors found that EPA reduced expression of immune response-related genes and boosted that of genes that combat oxidative stress. Many anti-inflammatory (and antioxidant) actions were specific to EPA and were not seen with oleic acid or palmitic acid, or control comparators. The novel observations from this paper greatly advance our understanding of the multitude of biological actions of EPA.

The transcriptomic analysis showed that EPA may prevent CD4+ T cell activation and subsequent differentiation by decreased transcription of genes critical to these T cell processes, including CD69 and IL2RA, respectively.^6^ Such changes occurred concomitant with decreased expression of transcription factors implicated in T cell differentiation, indicating a robust effect of EPA on these cells. Of particular interest, the transcriptomic analysis of nonactivated T cells revealed that EPA treatment modulated genes whose protein products were similarly expressed in other tissues, including human endothelial cells, indicating the presence of conserved mechanisms of action with EPA across multiple cell types.^6^^,^^7^ In both T cells and endothelial cells, EPA treatment specifically increased transcription of *HMOX1* and *NQO1*, which encode for the proteins heme oxygenase-1 (HO-1) and NAD(P)H quinone dehydrogenase 1 (NQO1), respectively.^6^^,^^7^ These genes are among those located within a nuclear factor erythroid 2-related factor 2 (NRF2)-dependent antioxidant response element that controls DNA transcription. In endothelial cells, EPA treatment significantly increased both HO-1 and NQO1 following IL-6 challenge.^7^ EPA enhances NRF2 translocation to the nucleus followed by transcription (and subsequent translation) of the cassette of genes influenced by the antioxidant response element. Thus, these new transcriptomic findings support systemic effects of EPA on particular pathways involved in the regulation of immune function, as previously reported in the vascular endothelium.^6^^,^^7^

Endothelial dysfunction and inflammation drive atherosclerosis and, if uninterrupted, can culminate with plaque rupture and thrombosis (Figure 1). A growing body of basic research, such as these T cell transcriptomic data, adds to our understanding of how EPA may modify atherosclerosis at multiple points to contribute mechanistically to the overall cardiovascular risk reduction noted in the outcome trials. Beyond reductions in inflammation and T cell activation, EPA improves endothelial vasodilator function and nitric oxide bioavailability while interrupting cholesterol crystallization and lipid oxidation even compared with other long chain fatty acids.^8^

**Figure 1:**
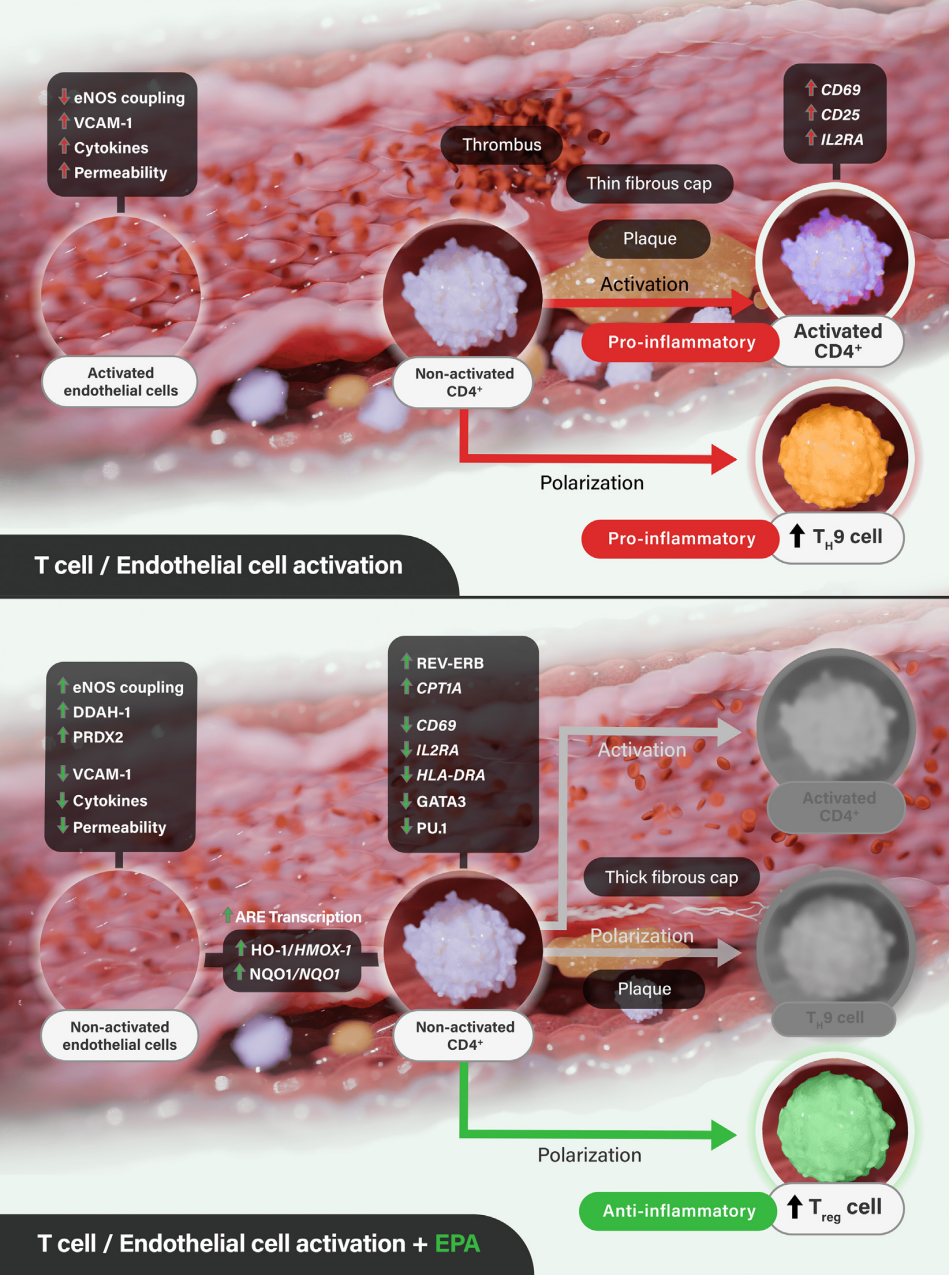
Emerging Pathways of Action of EPA Eicosapentaenoic acid (EPA) promotes broad anti-inflammatory effects in T cells and endothelial cells through directed changes in gene and protein expression that may limit progression of atherosclerosis beyond triglyceride lowering. When activated by binding to antigens, T cells release inflammatory signals that promote atherosclerosis in a subset-dependent manner. Following activation through toll-like receptors, naïve CD4⁺ T cells polarize to distinct cell populations with release of various cytokines and signaling molecules such as CD69, CD25, and interleukin 2 receptor subunit alpha (IL2RA). Activated cells express CD40L that assist the function of B cells and other immune cells. In parallel fashion, activated endothelial cells express adhesion molecules and release proinflammatory factors, along with increased vascular permeability and reduced nitric oxide (NO) release. Reilly et al^6^ reported that EPA treatment induces anti-inflammatory changes in T cell transcriptomes that complement proteomic findings in vascular endothelial cells.^6^ In both T cells and endothelial cells, EPA increased expression and release of cytoprotective proteins such as HO-1/*HMOX1*, NAD(P)H dehydrogenase [quinone] 1 (NQO1/*NQO1*), and antioxidant response element (ARE) transcription products. Separately, EPA reduced transcription in nonactivated CD4⁺ T cells of *CD69*, *IL2RA*, major histocompatibility complex, class II, DR alpha (*HLA-DRA*), GATA binding protein 3 (GATA3), purine-rich box1 (PU.1) while increasing levels of carnitine palmitoyltransferase 1A (*CPT1A*) and REV-ERB, all of which indicate a reduction in T cell activation of polarization toward proinflammatory subsets and increased polarization to anti-inflammatory subsets including T_reg_. Under conditions of inflammation, EPA improves endothelial NO synthase (eNOS) coupling and bioavailability with increased expression of dimethylarginine dimethylaminohydrolase 1 (DDAH-1) and the detoxification protein peroxiredoxin-2 (PRDX2), while reducing expression of vascular cell adhesion protein 1 (VCAM-1), cytokines, and vascular permeability. Other actions of EPA include antithrombotic effects, cell-membrane stabilizing properties, and reduction of plaque progression.

Inflammation contributes centrally to the genesis of plaque rupture and ischemic events. Contemporary data support that the risk associated with arterial inflammation persists independently of low-density lipoprotein cholesterol and triglyceride levels.^9^ The data from Reilly et al^6^ suggest that EPA could help address this component of residual cardiovascular risk. Analyses from REDUCE-IT show consistent clinical benefits of EPA across baseline levels of low-density lipoprotein cholesterol (and triglycerides, as noted in the previous text). Anti-inflammatory effects mediated by the adaptive immune modulation beyond those assessed by the biomarkers of innate immunity previously reported in REDUCE-IT, such as the effects on T cells reported by Reilly et al,^6^ could contribute to these benefits.

The valuable data from Reilly et al^6^ add to other important experimental data that have shown cell membrane stabilizing properties, other anti-inflammatory effects, antioxidant benefits, and antithrombotic properties of EPA, beyond its ability to lower levels of triglyceride-rich lipoproteins.^8^ These laboratory data complement several randomized imaging trials that also show effects of EPA on slowing or even reversing coronary plaque progression, including the first demonstration of an effect of any drug on improving noninvasively determined fractional flow reserve on CT angiography.^10^

The present analysis should prompt further research on EPA. This molecule, highly conserved through evolution, appears to play a fundamental role in cardiovascular biology. In REDUCE-IT, no apparent excess in infections emerged; thus, unlike some other targeted anti-inflammatory agents, there does not appear to be an infectious disease risk associated with EPA. This observation opens the door to the study of EPA in other disease states where the effects described by Reilly et al^6^ on T cells may also provide clinical benefit.

Carefully performed basic science experiments such as the current ones by Reilly et al^6^ should help convince physicians that data from REDUCE-IT and several other consistent clinical and imaging trials have a firm biological basis. Hopefully, this deeper understanding of the emerging pathways of action of EPA (Figure 1) will translate into appropriate patients being identified and treated with icosapent ethyl in daily practice.

## Funding Support and Author Disclosures

Dr Bhatt has served on the Advisory Board of Angiowave, Bayer, Boehringer Ingelheim, CellProthera, Cereno Scientific, Elsevier Practice Update Cardiology, High Enroll, Janssen, Level Ex, McKinsey, Medscape Cardiology, Merck, MyoKardia, NirvaMed, Novo Nordisk, PhaseBio, PLx Pharma, and Stasys; has served on the Board of Directors of American Heart Association New York City, Angiowave (stock options), Bristol Myers Squibb (stock), DRS.LINQ (stock options), and High Enroll (stock); has served as a consultant for Broadview Ventures, GlaxoSmithKline, Hims, SFJ, and Youngene; has served on Data Monitoring Committees for Acesion Pharma, Assistance Publique-Hôpitaux de Paris, Baim Institute for Clinical Research (formerly Harvard Clinical Research Institute, for the PORTICO trial, funded by St. Jude Medical, now Abbott), Boston Scientific (Chair, PEITHO trial), Cleveland Clinic, Contego Medical (Chair, PERFORMANCE 2), Duke Clinical Research Institute, Mayo Clinic, Mount Sinai School of Medicine (for the ENVISAGE trial, funded by Daiichi Sankyo; for the ABILITY-DM trial, funded by Concept Medical; for ALLAY-HF, funded by Alleviant Medical), Novartis, Population Health Research Institute; and Rutgers University (for the National Institutes of Health-funded MINT Trial); has received honoraria from the American College of Cardiology (Senior Associate Editor, Clinical Trials and News, ACC.org; Chair, ACC Accreditation Oversight Committee), Arnold and Porter law firm (work related to Sanofi/Bristol-Myers Squibb clopidogrel litigation), Baim Institute for Clinical Research (formerly Harvard Clinical Research Institute; RE-DUAL PCI clinical trial steering committee funded by Boehringer Ingelheim; AEGIS-II executive committee funded by CSL Behring), Belvoir Publications (Editor-in-Chief, Harvard Heart Letter), Canadian Medical and Surgical Knowledge Translation Research Group (clinical trial steering committees), CSL Behring (AHA lecture), Cowen and Company, Duke Clinical Research Institute (clinical trial steering committees, including for the PRONOUNCE trial, funded by Ferring Pharmaceuticals), HMP Global (Editor in Chief, Journal of Invasive Cardiology), Journal of the American College of Cardiology (Guest Editor; Associate Editor), K2P (Co-Chair, interdisciplinary curriculum), Level Ex, Medtelligence/ReachMD (CME steering committees), MJH Life Sciences, Oakstone CME (Course Director, Comprehensive Review of Interventional Cardiology), Piper Sandler, Population Health Research Institute (for the COMPASS operations committee, publications committee, steering committee, and USA national co-leader, funded by Bayer), WebMD (CME steering committees), and Wiley (steering committee); has served as Deputy Editor of Clinical Cardiology; is named on a patent for sotagliflozin assigned to Brigham and Women's Hospital who assigned to Lexicon (neither he nor Brigham and Women's Hospital receive any income from this patent); has received research funding from Abbott, Acesion Pharma, Afimmune, Aker Biomarine, Alnylam, Amarin, Amgen, AstraZeneca, Bayer, Beren, Boehringer Ingelheim, Boston Scientific, Bristol-Myers Squibb, Cardax, CellProthera, Cereno Scientific, Chiesi, CinCor, Cleerly, CSL Behring, Eisai, Ethicon, Faraday Pharmaceuticals, Ferring Pharmaceuticals, Forest Laboratories, Fractyl, Garmin, HLS Therapeutics, Idorsia, Ironwood, Ischemix, Janssen, Javelin, Lexicon, Lilly, Medtronic, Merck, Moderna, MyoKardia, NirvaMed, Novartis, Novo Nordisk, Otsuka, Owkin, Pfizer, PhaseBio, PLx Pharma, Recardio, Regeneron, Reid Hoffman Foundation, Roche, Sanofi, Stasys, Synaptic, The Medicines Company, Youngene, and 89Bio; has received royalties from Elsevier (Editor, Braunwald’s Heart Disease); has served as Site Co-Investigator for Abbott, Biotronik, Boston Scientific, CSI, Endotronix, St. Jude Medical (now Abbott), Philips, SpectraWAVE, Svelte, and Vascular Solutions; is a Trustee of the American College of Cardiology; and has performed unfunded research for FlowCo. Dr Libby is an unpaid consultant to or involved in clinical trials for Amgen, AstraZeneca, Baim Institute, Beren Therapeutics, Esperion Therapeutics, Genentech, Kancera, Kowa Pharmaceuticals, Merck, Moderna, Novo Nordisk, Novartis, Pfizer, and Sanofi-Regeneron; is a member of the scientific advisory board for Abcentra, Amgen, Caristo Diagnostics, Cartesian Therapeutics, CSL Behring, Elucid Bioimaging, Kancera, Kowa Pharmaceuticals, Olatec Therapeutics, Medimmune, Novartis, PlaqueTec, Polygon, TenSixteen Bio, Soley Therapeutics, and XBiotech, Inc; his laboratory has received research funding in the last 2 years from Novartis, Novo Nordisk and Genentech; is on the Board of Directors of and has a financial interest in XBiotech, a company developing therapeutic human antibodies; has a financial interest in TenSixteen Bio, a company targeting somatic mosaicism and clonal hematopoiesis of indeterminate potential (CHIP) to discover and develop novel therapeutics to treat age-related diseases, and in Soley Therapeutics, a biotechnology company that is combining artificial intelligence with molecular and cellular response detection for discovering and developing new drugs, currently focusing on cancer therapeutics; and receives funding support from the National Heart, Lung, and Blood Institute (1R01HL134892 and 1R01HL163099-01), the RRM Charitable Fund, and the Simard Fund; his interests were reviewed and are managed by Brigham and Women’s Hospital and Mass General Brigham in accordance with their conflict-of-interest policies. Dr Mason has received consulting or research grants from Amarin Pharma Inc, HLS Therapeutics, Esperion, Lexicon, and Cleveland Clinic.
